# Surveys in the Chrysanthemum Production Areas of Brazil and Colombia Reveal That Weeds Are Potential Reservoirs of Chrysanthemum Stunt Viroid

**DOI:** 10.3390/v11040355

**Published:** 2019-04-17

**Authors:** Danielle Gobatto, Lucas Araújo de Oliveira, Daniel Andrade de Siqueira Franco, Nubia Velásquez, José-Antonio Daròs, Marcelo Eiras

**Affiliations:** 1 Lab. Fitovirologia e Fisiopatologia, Centro de Pesquisa de Sanidade Vegetal, Instituto Biológico, São Paulo, SP 04014-002, Brazil; daniellegobatto@gmail.com (D.G.); araujo.oliveira.adm@gmail.com (L.A.d.O.); 2 Centro Avançado de Pesquisa em Proteção de Plantas e Saúde Animal, Instituto Biológico, Campinas, SP 13101-680, Brazil; franco@biologico.sp.gov.br; 3 Universidad Católica de Oriente, Grupo de Investigación de Sanidad Vegetal (GISAVE), Sector 3, cra. 46 No. 40B 50, Rionegro, Colombia; nubiayin@gmail.com; 4 IBMCP (CSIC-Universitat Politècnica de València), 46022 Valencia, Spain; jadaros@ibmcp.upv.es

**Keywords:** CSVd, *Pospiviroid*, *Pospiviroidae*, viroid reservoir

## Abstract

The stunting disease, incited by chrysanthemum stunt viroid (CSVd), has become a serious problem in chrysanthemum production areas worldwide. Here we identified 46 weed species from chrysanthemum fields in two producing regions of the State of São Paulo, Brazil. The mechanical inoculation of these weeds with a Brazilian CSVd isolate revealed that this viroid was able to infect 17 of these species, in addition to chrysanthemum, tomato and potato. Plants of *Oxalis latifolia* and chrysanthemum naturally infected with CSVd were found in chrysanthemum fields in Colombia, which is the first CSVd report in that country. Therefore, weeds have the potential to act as reservoirs of CSVd in the field. These results are the first reports of experimental CSVd infection in the following species: *Amaranthus viridis*, *Cardamine bonariensis*, *Chamaesyce hirta*, *Conyza bonariensis*, *Digitaria sanguinalis*, *Gomphrena globosa*, *Helianthus annuus*, *Lupinus polyphyllus*, *Mirabilis jalapa*, *Oxalis latifolia*, *Portulaca oleracea* and *Catharanthus roseus*. The phylogenetic analyses of the CSVd variants identified herein showed three groups with Brazilian CSVd variants distributed in them all, which suggests that Brazilian CSVd isolates may have different origins through successive introductions of infected germplasm of chrysanthemum in Brazil.

## 1. Introduction

Viroids consist of naked infectious, covalently closed circular, single-stranded, non-coding RNAs of 245–401 nucleotides in size. They are totally dependent on enzymes and other cell host factors to complete all steps of their infectious cycle, including replication, processing, movement and pathogenesis [1,2,3,4,5,6]. Restricted to higher plants, they can infect monocot and dicot hosts, including economically important herbaceous plants like cucumber, hop, potato and tomato, and ornamental plants like chrysanthemum, dahlia or coleus. They also infect woody perennials like apple, avocado, citrus, coconut palm, grapevine, peach, pear and plum [7]. These infections can cause important diseases by inciting severe symptoms that lead to major economic loss. However, in many situations, viroid infections occur by inducing mild symptoms or not inducing presumptive symptoms, which facilitates the maintenance of the infectious agent in the field and its spread through the exchange of infected plants and vegetative propagation materials [8,9,10,11].

Viroid species are classified by the International Committee on Virus Taxonomy (ICTV) in the Realm *Riboviria* [12] into two families: *Avsunviroidae*, which is composed of three genera (*Avsunviroid*, *Pelamoviroid* and *Elaviroid*), and *Pospiviroidae*, with five genera (*Pospiviroid*, *Hostuviroid*, *Cocadviroid*, *Apscaviroid* and *Coleviroid*). Members belonging to *Avsunviroidae*, like its type species *Avocado sunblotch viroid* (Genus *Avsunviroid*), replicate in plastids through a symmetric rolling-circle mechanism, and their RNAs self-cleave by the activity of internal hammerhead ribozymes. *Potato spindle tuber viroid* (Genus *Pospiviroid*) is the type species of *Pospiviroidae*. Members of this family have five structural/functional domains, including a characteristic central conserved region (CCR), and frequently adopt a rod-shaped secondary structure. They replicate and accumulate in the nuclei of infected cells following an asymmetric rolling-circle mechanism and they lack ribozyme activity [7,13].

Since the discovery and proposition of the term viroid as a new biological entity for potato spindle tuber viroid (PSTVd), the causal agent of “potato spindle tuber” disease [14,15,16], and for citrus exocortis viroid (CEVd), the causal agent of “citrus exocortis” [17,18], a number of other diseases of undetermined etiology have been confirmed to be induced by viroids [7,19]. “Chrysanthemum stunt disease”, incited by chrysanthemum stunt viroid (CSVd, genus *Pospiviroid*, family *Pospiviroidae*), was one of them. This disease was reported for the first time in the 1940s in the USA [20]. Almost 30 years later in the 1970s, its viroid etiology was identified [21,22,23,24,25,26]. With a worldwide distribution and occurrence confirmed in at least 26 countries, stunting disease has become a serious problem for chrysanthemum (*Dendranthema* spp., formerly *Chrysanthemum* spp.) production systems [8,27,28]. The main countries exporting cut flowers, including chrysanthemum, are The Netherlands (52%), followed by Colombia (35%) and Ecuador (9%) [29,30]. Brazil is one of the largest consumers of ornamental flowers, and 97% of its supply comes from its domestic production. Led by chrysanthemum, the production and marketing of ornamentals consists of a successful Brazilian agribusiness branch that moves about two billion US dollars per year. Chrysanthemum stunt disease is considered one of the main challenges for chrysanthemum production in all these countries [8,27,28].

In addition to stunting, CSVd can also incite, in some chrysanthemum-susceptible cultivars, color breaking, reduced flower size, delayed flowering and poor root development, which affect their quality and quantity, and hinder their marketing [11,28,31,32]. Notably, CSVd does not induce visible symptoms (latency) in many situations, with cases of tolerant/resistant cultivars, together with its transmission by vegetative propagation, easy mechanical transmission and wide host range, seems to facilitate its spread in the field by it passing international borders unnoticed [8,11,27,28,33,34].

In addition to cultivated and several wild species of chrysanthemum, CSVd has been found to naturally infect *Ageratum* spp., *Argyranthemum frutescens*, *Dahlia* spp., *Petunia* hybrida, *Solanum jasminoides* and *Vinca major* [28,35,36,37,38,39,40]. The experimental host range of CSVd includes a list of more than 50 species from different botanical families, as well as vegetable species such as cucumber (*Cucumis sativus*), sweet pepper (*Capsicum annuum*), eggplant (*Solanum melongena*) and tomato (*S. lycopersicum*), and weeds like *Emilia sagittata*, *Nicandra physalodes* and *Sonchus asper* [28,35,36,41]. Most of these infections occur with no symptom expression. Worse still, some discrepancy exists in the literature as to CSVd hosts, which leads to conflicting conclusions [28,31].

Due to its contagious nature, the long latent period and the vegetative propagation of the chrysanthemum, the control measures of CSVd, listed as A2 quarantine pest by the European and Mediterranean Plant Protection Organisation [42], are based on the implementation of preventive strategies to avoid transmission by hands, tools and plant-to-plant contact. Other measures can be taken, such as obtaining viroid-free germplasm, removing sources of infectious materials and blocking viroid entry to the seedling production system by indexing phytosanitary programs supported by reliable diagnostic methods [8,31,43,44,45]. Moreover, strategies for managing stunt disease include natural or transgenic resistance [46,47,48], and anti-viroid agents [49].

Weeds can apparently act as natural reservoirs of CSVd in the field, and may significantly contribute to maintain the inoculum source of the disease in chrysanthemum-cultivated areas [50]. To evaluate this problem in two major chrysanthemum-producing areas in South America, chrysanthemums and weeds associated with this crop were surveyed. It was found that the weed *Oxalis latifolia* is a CSVd reservoir in Colombia and up to 17 weed species in Brazil were identified as hosts of CSVd and can, consequently, act as viroid reservoirs.

## 2. Materials and Methods

### 2.1. Phytosociological Survey in Fields of Vase and Cut Chrysanthemums

**Figure 1:**
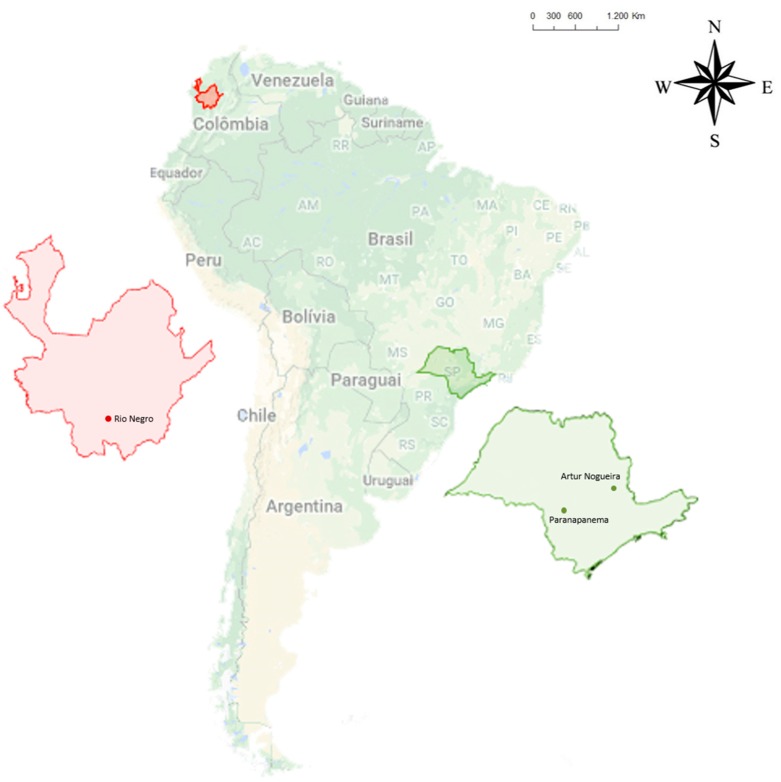
Major chrysanthemum-producing regions in the state of São Paulo, Brazil, and in Rionegro, Antioquia, Colombia, where different chrysanthemum cultivars and associated weeds were surveyed for chrysanthemum stunt viroid (CSVd) infection.

Plants of several chrysanthemum cultivars and weeds of different species were collected in chrysanthemum fields from two producers in the regions of Artur Nogueira and Paranapanema, São Paulo (Brazil), and in seven producers located in Rionegro, Antioquia (Colombia) (Figure 1). Collection was based on the method described by Braun-Blanquet [51], with modifications, using a marker (square) of 1 m^2^ randomly placed in selected areas. According to the described method, the number of entries in the quadrant depends on the size and type of the analyzed area. The fields selected for this study produced both pot and greenhouse chrysanthemums, and a uniform collection system was maintained for them all. The weed collection system was carried out as follows: inside greenhouses, around them and between chrysanthemum production lines. Whole plants containing roots and seeds were arranged in pots that could hold 350 mL of substrate kept in greenhouses. After the seed germination period, all the plants were subjected to molecular tests to evaluate the presence or absence of CSVd. The plants scored as being negative for viroid infection were challenged by mechanical inoculation by rubbing an extract from young leaves of the chrysanthemums infected with a Brazilian CSVd isolate [8]. Plants were retested 60 days post-inoculation by Reverse Transcription Polymerase Chain Reaction (RT-PCR) to evaluate the presence or absence of CSVd.

### 2.2. Mechanical Inoculation and Experimental Host Range

The weeds usually found in chrysanthemum-producing fields in Brazil, plant species reported as host indicators of CSVd [52], and some ornamental species cultivated in Brazil [29,30], were selected to evaluate the (experimental) host range for a previously characterized Brazilian CSVd isolate (2214-BR, Genbank accession number JX909290) [8]. The following species, belonging to 16 botanical families, were challenged: *Amaranthus viridis*, *Gomphrena globosa* (Amaranthaceae), *Catharanthus roseus* (Apocynaceae), *Ageratum conyzoides*, *Bidens pilosa*, *Chrysanthemum grandiflora*, *Chrysanthemum carinatum*, *Emilia sagittata*, *Helianthus annuus*, *Melampodium perfoliatum*, *Senecio cruentus*, *Sonchus oleraceus, Xanthium strumarium*, *Zinnia elegans* (Asteraceae), *Brassica napus*, *Raphanus raphanistrum* (Brassicaceae), *Lobelia erinus* (Campanulaceae), *Gypsophila elegans* (Caryophyllaceae), *Hemiscola aculeata* (Cleomaceae), *Arachis pintoi*, *Cassia occidentalis*, *Lupinus polyphyllus* (Fabaceae), *Mirabilis jalapa* (Nyctaginaceae), *Oxalis corniculata*, *O. latifolia* (Oxalidaceae), *Eschscholzia californica* (Papaveraceae), *Antirrhinum majus* (Plantaginaceae), *Portulaca grandiflora*, *P. oleracea* (Portulacaceae), *Fragaria vesca* (Rosaceae), *Ruta graveolens* (Rutaceae), *Nicandra physalodes*, *Nicotiana benthamiana*, *N. megalosiphon*, *N. occidentalis*, *N. tabacum*, *N. rustica*, *Solanum americanum*, *S. lycopersicum*, *S. tuberosum* (Solanaceae) and *Viola tricolor* (Violaceae). The seeds of these plants were arranged in trays containing substrate and were transplanted approximately 15 days after germination in vessels that could hold 350 mL. Five vessels were selected, where the plants of four of these vessels were subjected to mechanical inoculation 10 days after the transplantations. The selected plants were inoculated with plant extract from young leaves of the chrysanthemums infected with CSVd. Extracts were obtained by trituration in a pre-sterilized mortar and pestle in the presence of 0.5% sodium sulfite solution, at pH 6.0 and a ratio of 1:5 (g:mL) silicon carbide as the abrasive. Inoculations were carried out by rubbing the plant infectious extract with a porcelain pestle on the adaxial surface of leaves. The control plants were mock-inoculated with only 0.5% sodium sulfite solution, pH 6.0. Plants were washed with water and kept in a greenhouse to observe symptoms. Leaves were collected for the total RNA purification and RT-PCR analyses 60 days after inoculation.

### 2.3. RNA Purification for the RT-PCR Analysis

RNAs were purified according to the protocol described by Salzman et al. [53] with modifications. Leaf samples (0.1 g) were macerated in a previously sterilized mortar in the presence of liquid nitrogen. Then 0.5 mL of extraction buffer was added and homogenization continued. The extraction buffer consisted of 160 μL of 2-mercaptoethanol, 0.08 g of polyvinylpyrrolidone (PVP) and 8 mL of solution D (4 M guanidine isothiocyanate, 10% sarcosyl, 0.75 M sodium acetate and sterile water for a final volume to 500 mL). After homogenizing for 1 min, 0.8 mL of a mix of chloroform and isoamyl alcohol (24:1) was added and the mixture was homogenized for 20 min. Contents were transferred to 2 mL tubes and centrifuged at 13,000 rpm for 10 min at 4 °C. The supernatant was transferred to a new 2 mL tube to which 0.8 mL chloroform and isoamyl alcohol (24:1) was added to be homogenized for 10 min, and centrifuged at 13,000 rpm for 10 min at 4 °C. Once again, the supernatant was transferred to new tubes. Two volumes of ethanol and a 0.1 volume of NaCl (5 M) were added to precipitate nucleic acids at −20 °C for 12 h. After this period, tubes were centrifuged at 13,000 rpm for 10 min at 4 °C and the supernatant was discarded. Sediments were resuspended with 0.8 mL of sterile water. A solution of phenol:chloroform:isoamyl alcohol (25:24:1) was added, and the mixture was homogenized at room temperature for 5 min. Tubes were centrifuged at 10,000 rpm for 10 min at 15 °C and the supernatant was transferred to new tubes. Nucleic acids were precipitated at −20 °C for 24 h in the presence of two volumes of ethanol and a 0.1 volume of 5 M NaCl. Tubes were recentrifuged at 13,000 rpm for 10 min at 4 °C, the supernatant was discarded, and the pellet was resuspended in 10 μL of sterile water. RNAs were stored at −20 °C.

### 2.4. RNA Purification for Sequential Polyacrylamide Gel Electrophoresis (sPAGE)

The leaves (5 g) of chrysanthemums and weeds were ground in liquid nitrogen and homogenized in the presence of a water-saturated phenol and extraction buffer mixture (125 mM Tris-HCl, pH 9.0, 0.75% sodium dodecyl sulfate (SDS), 15 mM ethylenediamine tetraacetic acid, (EDTA), 100 mM 2-mercaptoethanol). Total RNA was fractionated by chromatography on nonionic cellulose CF11 (Whatman, UK), recovered by ethanol precipitation and resuspended in sterile water. Aliquots of the RNA preparations were electrophoresed in a nondenaturing 5% polyacrylamide gel stained with ethidium bromide. A segment of this gel, delimited by the purified RNAs of avocado sunblotch viroid (ASBVd, 246 nt) and citrus exocortis viroid (CEVd, 371 nt), was cut and applied onto a second denaturing 5% polyacrylamide gel which, after electrophoresing, was stained with silver nitrate [8].

### 2.5. RT-PCR

For the cDNA synthesis, purified RNAs (*ca*. 100 ng) were transferred to a sterile microtube in the presence of 1 µL (50 pmol/µL) of the complementary primer (CSVd(c) 5′ GGGGATCCCTGAAGGACTTCT 3′), designed to anneal specifically in the CCR of the CSVd genome. After a 3-min incubation at 95 °C, 2.5 µL of reverse transcriptase buffer (Roche, Basel, Switzerland), 0.5 µL dNTPs (10 mM), 1 µL (200 U) of reverse transcriptase enzyme (Roche, Switzerland) were added, and the mixture was incubated for 60 min at 37 °C. PCR was carried out in a 50 µL volume using 1 µL of the cDNA product, 2.5 U of *Tth* DNA polymerase (Biotools, Madrid, Spain), 50 pmol of each primer (CSVd(c) and CSVd(s) 5′ GGGGAAACCTGGAGGAAG 3′) [54], 0.5 µL dNTPs and the buffer recommended by the supplier. Samples were denatured at 94 °C for 2 min, and the reaction conditions consisted of 30 cycles of 40 s at 94 °C, 30 s at 60 °C, and 2 min at 72 °C, with a final 10-min extension step at 72 °C. The amplified DNA fragments (with an expected size from 354 to 356 bp) were visualized in the agarose gels stained with ethidium bromide under ultraviolet irradiation.

### 2.6. Sequencing, Sequence Analyses and Phylogeny

The full-length amplification products were eluted from the gel using the Concert™ Rapid Gel kit (Gibco BRL™, Rochville, MD, USA), before being precipitated and resuspended in sterile water following the manufacturer’s instructions. The purified DNA products were subjected to direct sequencing according to the chain termination method [55] using the BigDye kit (Applied Biosystems, Foster City, CA, USA) and were sequenced in an ABI PRISM 377 DNA Sequencer (Applied Biosystems, Foster City, CA, USA) following the manufacturer’s instructions. Comparisons with other sequences in databases were made with the Basic Local Alignment Search Tool, BLASTn program (National Center for Biotechnology Information, NCBI), available on the world wide web. Multiple alignments were conducted using the BioEdit 7.2.0. software (Carlsbad, CA, USA), and the RNA secondary structures were predicted with the Mfold program for circular molecules [56]. Phylogenetic trees were constructed by neighbor joining (NJ) using the MEGA 7.0 software. Tree branches were tested by bootstrap with 1500 replications.

## 3. Results and Discussion

### 3.1. Detection of Chrysanthemum Stunt Viroid (CSVd) in Broadleaf Woodsorrel and Chrysanthemum in Colombia

**Figure 2:**
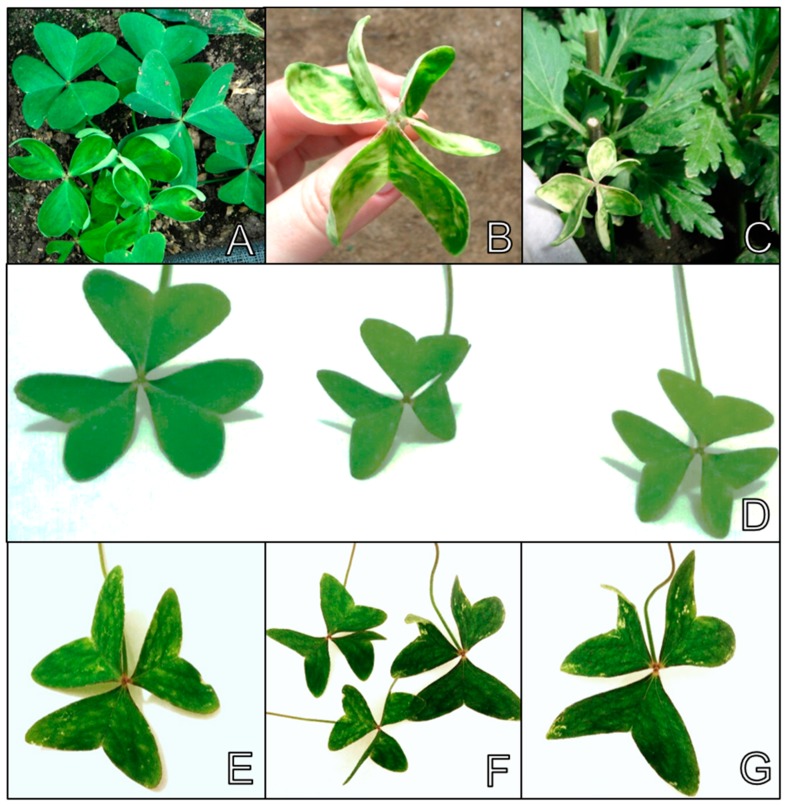
Broadleaf woodsorrel (*O. latifolia*) plants found in chrysanthemum fields in the Rionegro region, Antioquia (Colombia), showing mosaic and leaf distortions (**A**–**C**). Healthy broadleaf woodsorrel plants used in the inoculation experiments (**D**). Plants of broadleaf woodsorrel, 60 days after the inoculations with a Brazilian chrysanthemum stunt viroid (CSVd) isolate, showing symptoms of mosaic and leaf distortions (**E**–**G**).

*Oxalis latifolia* (broadleaf woodsorrel) is a weed with a worldwide distribution that is found in different cultivation types in orchards, nurseries, gardens and urban gardens as it easily adapts to tropical and temperate climates [57]. It is widely disseminated in chrysanthemum fields in Artur Nogueira and Paranapanema, Brazil, and also in Rionegro, Antioquia, Colombia. Originally from Mexico and belonging to the family Oxalidaceae, the genus *Oxalis* is the largest in the family with 800 species distributed worldwide. They are widely distributed in tropical and subtropical countries like Colombia and Brazil, and in countries in Africa and other regions in the Americas [57]. As this weed propagates vegetatively, there is an enormous potential for pathogens to disseminate, as viruses and viroids. In the chrysanthemum-producing fields in Rio Negro, Antioquia (Colombia), *O. latifolia* plants displayed mosaic and foliar deformation symptoms (Figure 2A–C). These plants were collected and subjected to an RT-PCR analysis with CSVd-specific oligonucleotides [54]. The chrysanthemums located around the symptomatic broadleaf woodsorrel plants were also collected and analyzed. Sequencing the amplified products confirmed the presence of CSVd in both symptomatic broadleaf woodsorrel and chrysanthemum plants. The sequence of the broadleaf woodsorrel Colombian CSVd isolate was deposited in Genbank under accession code MF359717, and the chrysanthemum isolates under accession codes MF359712 to MF359716. Note that in our analyses we used a non-proof-reading polymerase and a single pair of primers that covered the highly conserved CCR. It is noteworthy that the broadleaf woodsorrel plants in the chrysanthemum field in the Rio Negro region were naturally infected by CSVd. This finding indicates that this weed may play an important role as a reservoir and a potential source of viroid inoculum in the field. *Oxalis corniculata* (creeping woodsorrel) has been previously reported as a non-host species of CSVd [22].

### 3.2. Experimental Host Range of the Brazilian CSVd Isolate

After detecting that CSVd naturally infects weeds, which are common in chrysanthemum fields in Colombia, 56 plant species, which are frequent in Brazilian chrysanthemum fields, were first analyzed for the presence of CSVd and then challenged with a previously characterized Brazilian CSVd isolate [8]. No natural CSVd infection was detected in any of them. However, when inoculated with CSVd, systemic viroid infection was confirmed by RT-PCR (Figure S1) 60 days after inoculation in 17 of them: *A. viridis* (green amaranth), *C. bonariensis* (cardamine, a kind of cress), *C. roseus* (rose periwinkle), *C. hirta* (snakeweed), *C. bonariensis* (hairy fleabane), *C. carinatum* (tricolor daisy), *D. sanguinalis* (hairy crabgrass), *E. sagittata* (tasselflower), *G. globosa* (globe amaranth), *H. annuus* (sunflower), *L. polyphyllus* (lupine), *M. jalapa* (four-clock flower), *O. latifolia* (broadleaf woodsorrel or garden pink-sorrel), *P. oleracea* (common purslane), *S. cruentus* (cineraria), *S. lycopersicum* “Rutgers” (tomato), *S. tuberosum* “Agata” (potato) (Table 1).

Mosaic symptoms were observed only in *O. latifolia* (Figure 2), while dwarfism symptoms were noted in only four of the inoculated species, namely *A. viridis*, *E. sagittata*, *L. polyphyllus* and *M. jalapa*. For the remaining evaluated hosts that were susceptible to CSVd, infections occurred with no expressed symptoms.

In the chrysanthemum fields, in São Paulo, Brazil, the plants of broadleaf woodsorrel (*O. latifolia*) inside and around chrysanthemum greenhouses were collected. After the RT-PCR evaluations, they were all considered free of CSVd (Figure 2D). However, when the seedlings of this species were challenged by mechanical inoculations with a Brazilian CSVd isolate, the inoculated plants started showing symptoms of mosaic and foliar deformation 60 days after inoculations (Figure 2E–G), and CSVd infection was confirmed by RT-PCR (Figure S1). Interestingly, the infected broadleaf woodsorrel plants from Rio Negro, Antioquia (Colombia), showed the same mosaic and foliar deformation symptoms (Figure 2A–C), similarly to those observed in the plants of this same species inoculated with the Brazilian CSVd isolate.

**Figure 3:**
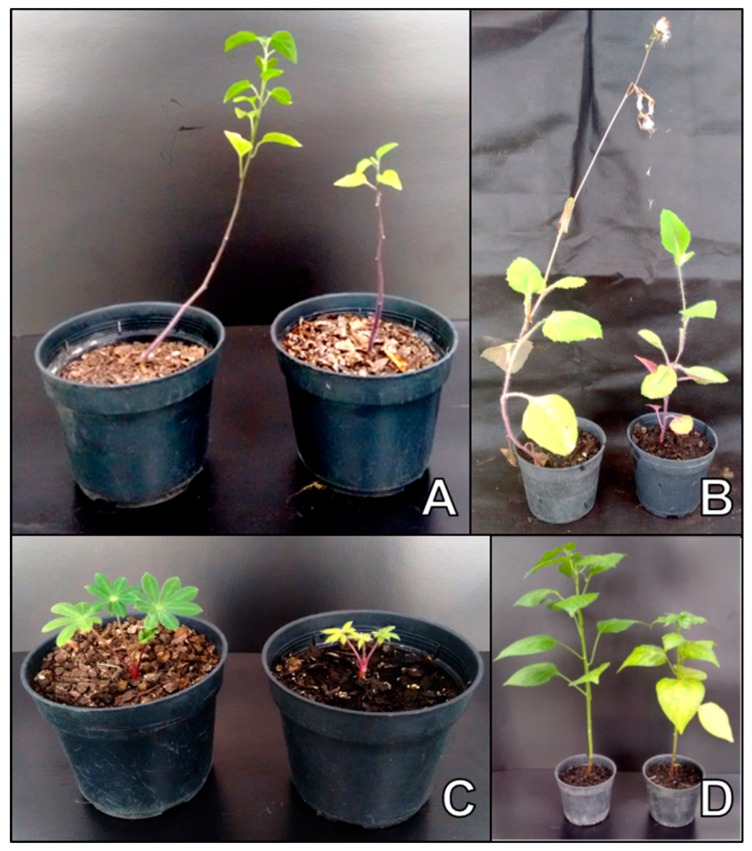
*Amaranthus viridis* (**A**), *Emilia sagittata* (**B**), *Lupinus polyphyllus* (**C**) and *Mirabilis jalapa* (**D**) showing dwarfism 60 days after inoculations with a Brazilian chrysanthemum stunt viroid (CSVd) isolate. The non-inoculated (healthy) controls are shown in each photo on the left.

*Amaranthus viridis* (green amaranth) is an annual herbaceous species that is native to the Americas and can be found all over Brazil. Despite being eaten in some regions, it is often considered a weed because it is spontaneous and has adapted to different Brazilian climate conditions. It is commonplace in the cultivation areas of certain crops like potato, tomato, banana, papaya, mango, passion fruit, orange and guava. This is, therefore, the first report of experimental *A. viridis* infection by CSVd showing dwarfism symptoms (Figure 3A). *A. caudatus* (velvet flower) has been previously reported as a non-host of CSVd [22].

Native to Asia, *E. sagittata* is a relatively common weed found throughout Brazil [57], and has also been reported as a reservoir for tospoviruses [58]. Symptoms of epinasty had already been observed in *Emilia sonchifolia* (lilac tasselflower) plants inoculated experimentally with a Brazilian CSVd isolate [8]. In this survey with the challenging plants of *E. sagittata*, the inoculated plants showed stunting compared to the mock-inoculated plants 60 days after inoculation (Figure 3B). *E. sagittata* has previously been described as an experimental host of CSVd [59]. These results revealed that the distinct plant species belonging to the same genus, and inoculated with the same CSVd isolate, can present different behaviors as regards the expression of symptoms. The opposite has also been confirmed as it is difficult to establish biological differences between CSVd isolates [33].

The infection of *L. polyphyllus* (lupine) by CSVd by inciting dwarfism (Figure 3C) also draws our attention because this plant, which originates from North America, is widely used in Brazil for nitrogen fixation, and is planted in extensive areas where it remains in the field for a few months, and may serve as a source of viroid inoculum. *Mirabilis jalapa* (four-clock flower) is an annual herbaceous species that can be found all over Brazil, either spontaneously or cultivated in gardens. It frequently occurs in humid shady places, and settles in areas occupied by olives, and also in domestic and commercial orchards. As it propagates easily through seeds and is difficult to control and manage, the Brazilian Ministry of Agriculture has issued a normative instruction, IN-SDA/MAPA 36/2010, which establishes suitable phytosanitary requirements to import seeds destined for its propagation. Its susceptibility to CSVd (Figure 3D) reinforces the need to better control the trade and exchange of the seedlings and seeds of this species.

The weed that was most frequently observed in all the visited chrysanthemum producers in both Brazil and Colombia was *Cardamine bonariensis*, Brassicaceae, a kind of cress. This annual herbaceous species is found in the Midwest, Northeast, Southeast and South Brazil. It is quite a common species in nurseries of seedlings and is found between the structures that maintain pots, between seedling production trays, and between chrysanthemum-cutting production lines. Evaluations of its incidence, made in 2006, during a phytosociological survey of *C. bonariensis* on the main chrysanthemum fields in São Paulo, led to chrysanthemum growers to pay more attention to infestation by this weed [60]. Originally from Europe, we suspect that it has arrived at Brazilian crops along with plant material and via informal transits of plants and ornamental seedlings. To date, reports on virus or viroids infecting cardamine are not available. Albeit experimental, the possibility of infection by CSVd draws attention to the potential of this host serving as a source of inoculum of this viroid in chrysanthemum-producing fields.

In addition to the weed species described in detail above, *C. hirta*, *C. bonariensis* and *D. sanguinalis*, albeit asymptomatic, were also infected and, according to our knowledge, correspond to the first reports of these hosts’ experimental infection by CSVd. Plants of *G. globosa*, *P. oleracea* and *C. roseus* were also infected by the Brazilian isolate of CSVd, and these results do not agree with those obtained by Hollings and Stone [22]. *Senecio cruentus* plants were also infected by the Brazilian CSVd isolate, which confirmed previously published data [59]. *Helianthus annuus* plants were also infected, which corresponds to the first report of experimental infection of this host by CSVd, but in the literature, the species *H. tuberosus* (sunroot or Jerusalem artichoke) is described as a non host of this viroid [22]. Although many other plant species, including cineraria [26] and petunia [41,59], have already been reported as CSVd experimental hosts, Verhoeven et al. [39] reported the first natural infection of petunia plants by CSVd. The plants that were co-infected by tobacco mosaic virus (TMV) and potato virus Y (PVY) exhibited mosaic and yellowing of leaves, which most likely resulted from the synergistic effect of virus and viroid infections.

To date, only *Ageratum* sp., *Dahlia* spp., *P. hybrida*, *A. frutescens*, *S. jasminoides* and *V. major*, as well as cultivated and wild species of chrysanthemum, have been reported as natural hosts of CSVd [20,21,38,39,40,61,62,63,64]. It is worth mentioning that a CSVd isolate has been reported to infect asymptomatic *A. frutescens* plants in Italy imported from Brazil [64], which indicates that this host can act as a reservoir of CSVd, mainly because symptoms are lacking.

In this survey, the identification of new host species of CSVd highlights the potential of weed species on viroid epidemiology in the field. From 2007 to 2012 in Australia, Mackie et al. [65] carried out a survey of weeds in tomato, pepper and sweet-pepper fields. At the time, different weed species (*Solanum nigrum*, *Datura leichhardtii*, *Conyza bonariensis*, *Physalis angulata*), including native Australian species (*Atriplex semilunaris*, *Rhagodia eremaea* and *Streptoglossa sp*.), were identified as naturally infected with PSTVd. These authors demonstrated that PSTVd was widely disseminated in commercial fields of Australian solanaceous plants, and that a number of weeds would act as reservoirs of the viroid.

### 3.3. Diversity of CSVd Isolates: Sequence Analyses and Phylogeny

In our phytosocio-ecological study, plants of different chrysanthemum cultivars were collected in both Brazil and Colombia. By molecular analyses, CSVd was identified in 27 chrysanthemum samples collected in Brazil (2215-BR to 2241-BR) and in five chrysanthemum samples collected in Colombia (2242-CO to 2246-CO), in addition to a sample of *O. latifolia* (2247-CO). The RT-PCR-amplified DNA (with CSVd-specific primers) was sequenced, and the sequences of these variants were aligned and compared to other sequences deposited in Genbank. The sequence lengths of the Brazilian and Colombian CSVd variants were 354 to 356 nt. When compared to other full CSVd genome sequences, percentages of nucleotide identity ranging from 92% to 100% were observed. The presence of CSVd by sequential polyacrylamide gel electrophoresis (sPAGE) was also confirmed for some of these chrysanthemum samples (Figures S2 and S3). The sequences obtained in this work were deposited in Genbank (Table 2).

**Figure 4:**
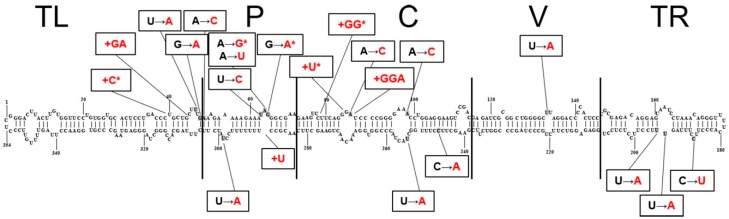
Predicted secondary structure of chrysanthemum stunt viroid (CSVd) Brazilian isolate 2214 (Genbank number JX909290) [8]. Changes (→) and insertions (+) in the residues among all Brazilian and Colombian CSVd variants were shown in the boxes. Changes observed in the Colombian variants were indicated with asterisks. The five domains of pospiviroids are marked: terminal left and right (TL and TR), pathogenicity (P), central conserved (C) and variable (V).

By the multiple alignment of the nucleotide sequences of the Brazilian and Colombian isolates with the CSVd sequence variants from other countries, identical sequences (Table 3) and mutations distributed throughout genomes were identified. The positions of the differences in residues among Brazilian and Colombian variants are shown in Figure 4. Most observed changes occurred in the upper strand of the molecule, mainly in regions not involved in base-pairing (loops). The sequence variants from Colombia were identical to one another, and were also identical to other Brazilian CSVd sequence variants (2234-BR, 2235-BR, 2236-BR, 2237-BR, 2238-BR, 2239-BR, 2240 -BR) from Artur Nogueira. The narrow variability observed in the CSVd isolates collected in Colombia can be explained by the fact that the collections were smaller and were concentrated in a single region, namely Rionegro. Very little genetic variability suggested that there had been fewer introductions of infected germplasm. It was also noted that the nucleotide sequence of the Colombian CSVd isolate from broadleaf woodsorrel (2247-CO) was identical to that of the chrysanthemum isolates, which suggests that this weed may have been a victim of viroid infection from chrysanthemum plants and served as a source of inoculum. Interestingly, the CSVd Brazilian isolate 2214 [8], used in mechanical inoculations, induced the same symptoms as the Colombian isolate 2247-CO in broadleaf woodsorrel plants. These isolates have 99% nucleotide identity, presenting only a change in the nucleotide 298 (U→A) (Figure 4).

The genetic variability and biological properties observed in both the Brazilian and Colombian isolates of the CSVd characterized herein can be compared to the results obtained with the CSVd isolates from the USA, China, Australia [33] and India [66] which, when inoculated in tomato plants and in chrysanthemum varieties, showed no differences in the expression of symptoms. The authors suggested that the biological variations could be due to both crop management and environmental effects. The CSVd genomes in Korea were highly conserved, but the authors revealed quasispecies of CSVd in single chrysanthemum plants [67].

**Figure 5:**
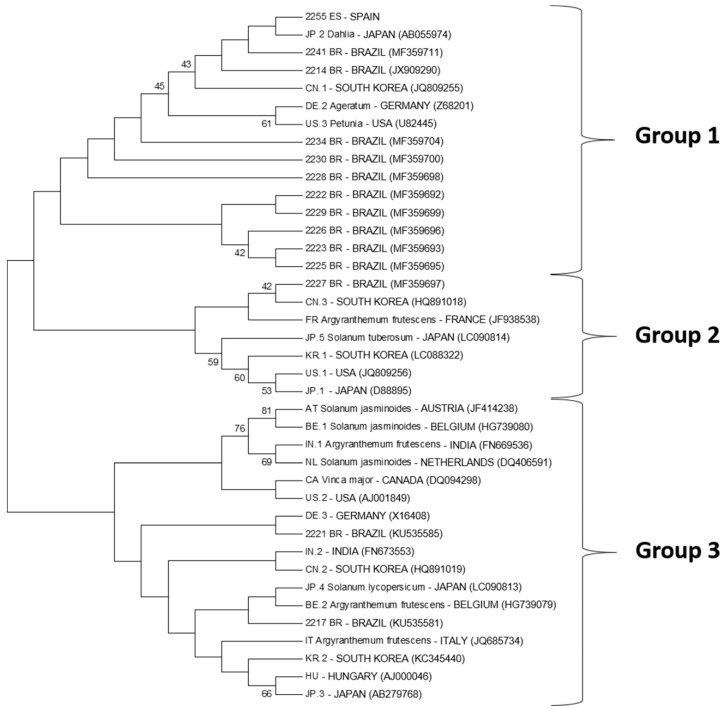
Unrooted phylogenetic tree based on the multiple alignment of the nucleotide sequences of the Brazilian and Colombian CSVd isolates, as well as a Spanish isolate (sequenced herein) and the sequence variants obtained from Genbank by the Neighbor Joining method (with Bootstrap analyses for 1500 replications) using the MEGA software (version 7.0) Three major groups (Group 1–3) were formed with the Brazilian CSVd isolates distributed in all the groups.

In the phylogenetic analyses carried out only with complete genome sequences, three major groups formed, with the Brazilian CSVd isolates distributed in all the groups, along with other CSVd sequence variants from Japan, China, Germany, France, South Korea, Austria, Belgium, India, The Netherlands, Canada, Italy and Hungary (Figure 5). It is noteworthy that the variants with identical sequences of both those deposited in Genbank and with those obtained herein were suppressed from the analyses, and only one sequence was maintained as a reference (Table 3). When analyzing CSVd variants in Korea, Choi et al. [67] also observed the formation of three phylogenetic groups. These results suggest that the Brazilian isolates of CSVd may have different origins, but with successive introductions of infected germplasms of chrysanthemum in Brazil.

## Figures and Tables

**Table 1:** Experimental chrysanthemum stunt viroid (CSVd) host range determined by mechanical inoculations with a Brazilian CSVd isolate. Infectivity was evaluated from the expression of symptoms and a RT-PCR analysis 60 days after inoculation.

Species/Family	Common Name	Infected/Inoculated Plants	RT-PCR	Symptoms *
*Aeschynomene rudis*/Fabaceae	Zigzag jointvetch	0/4	-	A
*Ageratum conyzoides*/Asteraceae	Billygoat-weed	0/4	-	A
*Amaranthus deflexus*/Amaranthaceae	Large-fruit amaranth	0/4	-	A
*A. viridis*/Amaranthaceae	Green amaranth	2/4	+	P (stunt)
*Antirrhinum majus*/Plantaginaceae	Snapdragon	0/4	-	A
*Arachis pintoi*/Fabaceae	Perennial Peanut	0/4	-	A
*Bidens pilosa*/Asteraceae	Hairy bidens	0/4	-	A
*Brassica napus*/Brassicacea	Summer rape	0/4	-	A
*Cardamine bonariensis*/Brassicacea	Cardamine (cress)	4/4	+	A
*Cassia occidentalis*/Fabaceae	Coffee senna	0/4	-	A
*Catharanthus roseus*/Apocynaceae	Rose periwinkle	3/4	+	A
*Chamaesyce hirta*/Euphorbiaceae	Snakeweed	3/4	+	A
*C. prostata*/Euphorbiaceae	Prostrate spurge	0/4	-	A
*Commelina benghalensis*/Commelinaceae	Benghal dayflower	0/4	-	A
*Conyza bonariensis*/Asteraceae	Hairy fleabane	4/4	+	A
*Chrysanthemum carinatum*/Asteraceae	Tricolor daisy	4/4	+	A
*Cyperus rotundus*/Cyperaceae	Nut Sedge	0/4	-	A
*Digitaria insularis*/Poaceae	Sourgrass	0/4	-	A
*D. sanguinalis*/Poaceae	Hairy crabgrass	4/4	+	A
*Eclipta prostrata*/Asteraceae	Eclipta	0/4	-	A
*Eleusine indica*/Poaceae	Indian goosegrass	0/4	-	A
*Emilia sagittata*/Asteraceae	Tasselflower	4/4	+	P (stunt)
*Eschscholzia californica*/Papaveraceae	California poppy	0/4	-	A
*Fragaria vesca*/Rosaceae	Woodland strawberry	0/4	-	A
*Gomphrena globosa*/Amaranthaceae	Globe Amaranth	4/4	+	A
*Godetia grandiflora*/Ericaceae	Clarkia amoena	0/4	-	A
*Gypsophila elegans*/Caryophyllaceae	Baby’s breath	0/4	-	A
*Helianthus annuus*/Asteraceae	Sunflower	3/4	+	A
*Hemiscola aculeata*/Cleomaceae	Prickly spider flower	0/4	-	A
*Lobelia erinus*/Campanulaceae	Lobelia	0/4	-	A
*Lupinus polyphyllus*/Fabaceae	Lupine	3/4	+	P (stunt)
*Melampodium perfoliatum*/Asteraceae	Blackfoots flower	0/4	-	A
*Mirabilis jalapa*/Nyctaginaceae	Four-clock flower	3/4	+	P (stunt)
*Nicandra physalodes*/Solanaceae	Shoo-fly plant	0/4	-	A
*Nicotiana benthamiana*/Solanaceae	Tobacco	0/4	-	A
*N. megalosiphon*/Solanaceae	Tobacco	0/4	-	A
*N. occidentalis*/Solanaceae	Nightshade	0/4	-	A
*N. rustica*/Solanaceae	Aztec tobacco	0/4	-	A
*N. tabacum* “Samsun”/Solanaceae	Tobacco	0/4	-	A
*N. tabacum* “Xanthi”/Solanaceae	Tobacco	0/4	-	A
*Oxalis corniculata*/Oxalidaceae	Creeping woodsorrel	0/4	-	A
*O. latifolia*/Oxalidaceae	Broadleaf woodsorrel	4/4	+	P (mosaic)
*Portulaca oleracea*/Portulacaceae	Common purslane	4/4	+	A
*Raphanus raphanistrum*/Brassicaceae	Wild radish	0/4	-	A
*Ruta graveolens*/Rutaceae	Rue	0/4	-	A
*Sagina chilensis*/Caryophyllaceae	Pearlworts	0/4	-	A
*Senecio cruentus*/Asteraceae	Cineraria	2/4	+	A
*Solanum americanum*/Solanaceae	Black nightshade	0/4	-	A
*S. lycopersicum* “Rutgers”/Solanaceae	Tomato	4/4	+	P (stunt)
*S. tuberosum* “Ágata”/Solanaceae	Potato	4/4	+	A
*Sonchus oleraceus*/Asteraceae	Sowthistle	0/4	-	A
*Tropaeolum majus*/Tropaeolaceae	Garden nasturtium	0/4	-	A
*Viola tricolor*/Violaceae	Johnny jump-ups	0/4	-	A
*Xanthium strumarium*/Asteraceae	Rough cocklebur	0/4	-	A
*Zinnia elegans*/Asteraceae	Common zinnia	0/4	-	A
*Z. elegans* “Lilliput”/Asteraceae	Zinnia	0/4	-	A

* A = absent; P = present; + = positive; - = negative.

**Table 2:** Sequence variants of chrysanthemum stunt viroid (CSVd) collected in two regions in the State of São Paulo, Brazil, and in the region of Rionegro, Antioquia, Colombia, with the access code in the Genbank and genome size.

Name	Origin	Genbank Code	Genome Size
2215-BR	Artur Nogueira, SP, Brazil	KU535579	354
2216-BR	Artur Nogueira, SP, Brazil	KU535580	354
2217-BR	Artur Nogueira, SP, Brazil	KU535581	354
2218-BR	Artur Nogueira, SP, Brazil	KU535582	354
2219-BR	Artur Nogueira, SP, Brazil	KU535583	354
2220-BR	Artur Nogueira, SP, Brazil	KU535584	354
2221-BR	Artur Nogueira, SP, Brazil	KU535585	354
2222-BR	Paranapanema, SP, Brazil	MF359692	354
2223-BR	Artur Nogueira, SP, Brazil	MF359693	355
2224-BR	Artur Nogueira, SP, Brazil	MF359694	354
2225-BR	Artur Nogueira, SP, Brazil	MF359695	356
2226-BR	Artur Nogueira, SP, Brazil	MF359696	356
2227-BR	Artur Nogueira, SP, Brazil	MF359697	354
2228-BR	Artur Nogueira, SP, Brazil	MF359698	354
2229-BR	Artur Nogueira, SP, Brazil	MF359699	354
2230-BR	Artur Nogueira, SP, Brazil	MF359700	356
2231-BR	Artur Nogueira, SP, Brazil	MF359701	354
2232-BR	Artur Nogueira, SP, Brazil	MF359702	354
2233-BR	Artur Nogueira, SP, Brazil	MF359703	354
2234-BR	Artur Nogueira, SP, Brazil	MF359704	354
2235-BR	Artur Nogueira, SP, Brazil	MF359705	354
2236-BR	Artur Nogueira, SP, Brazil	MF359706	354
2237-BR	Artur Nogueira, SP, Brazil	MF359707	354
2238-BR	Artur Nogueira, SP, Brazil	MF359708	354
2239-BR	Artur Nogueira, SP, Brazil	MF359709	354
2240-BR	Artur Nogueira, SP, Brazil	MF359710	354
2241-BR	Artur Nogueira, SP, Brazil	MF359711	354
2242-CO	Rionegro, Antioquia, Colombia	MF359712	354
2243-CO	Rionegro, Antioquia, Colombia	MF359713	354
2244-CO	Rionegro, Antioquia, Colombia	MF359714	354
2245-CO	Rionegro, Antioquia, Colombia	MF359715	354
2246-CO	Rionegro, Antioquia, Colombia	MF359716	354
2247-CO *	Rionegro, Antioquia, Colombia	MF359717	354

* Sequence of the CSVd variant isolated from *O. latifolia* (broadleaf woodsorrel) collected in chrysanthemum fields in Rionegro, Antioquia, Colombia.

**Table 3:** Sequence variants of chrysanthemum stunt viroid (CSVd) with identical nucleotide sequences to the variants deposited in Genbank.

Sequence Variants and Access Code in Genbank	Identical Sequence Variants
JP.2 (AB055974)	None
2241-BR (MF359711)	2248/2250/2251/2252/2253
2214-BR (JX909290)	None
CN.1 (JQ809255)	None
DE.2 (Z68201)	None
US.3 (U82445)	None
2234-BR (MF359704)	2235/2236/2237/2238/2239/2240/2242/2243/2244/2245/2246/2247
2230-BR (MF359700)	2231/2232
2228-BR (MF359698)	None
2222-BR (MF359706)	None
2229-BR (MF359699)	None
2226-BR (MF359696)	None
2223-BR (MF359693)	None
2225-BR (MF359695)	None
2227-BR (MF359697)	2218/2219/2220/2233
CN.3 (HQ891018)	CN.4
FR (JF938538)	None
JP.5 (LC090814)	None
KR.1 (LC088322)	None
US.1 (JQ895296)	US.3 a US.40
JP.1 (D88895)	JP.6/JP.7
AT (JF414238)	None
BE.1 (HG739080)	None
IN.1 (FN669536)	IN.3/IN.4/IN.5/IN.6
NL (DQ406591)	None
CA (DQ094298)	None
US.2 (AJ001849)	US.41 a US.64
DE.3 (X16408)	DE.1/DE.4
2221-BR (KU535585)	None
IN.2 (FN673553)	IN.7/IN.8/IN.9/IN.10/IN.11/IN.12/IN.13/IN.14
CN.2 (HQ891019)	Nenhum
JP.4 (LC090813)	JP.8/JP.9/JP.10/JP.11/JP.12
BE.2 (HG739079)	BE.3/BE.4
2217-BR (KU535581)	None
IT.1 (JQ685734)	IT.2/IT.3/IT.4/IT.5/IT.6/IT.7/IT.8/IT.9/IT.10/AU.1/AU.2/AU.3
KR.2 (KC345440)	KR.3/KR.4
HU (AJ000046)	None
JP.3 (AB279768)	JP.13/JP.14/JP.15/JP.16/JP.17
2255-ES (not deposited in Genbank)	None

## Supplementary Materials

The following are available online at https://www.mdpi.com/1999-4915/11/4/355/s1: Figure S1: RT-PCR analysis to confirm the infectivity of the Brazilian chrysanthemum stunt viroid (CSVd) isolate in several inoculated hosts. Figure S2. Sequential electrophoresis results on native and denaturing polyacrylamide gels (sPAGE) from the RNA samples extracted from the chrysanthemum plants collected in regions of Artur Nogueira, State of Sao Paulo, Brazil. Figure S3. Sequential electrophoresis results on native and denaturing polyacrylamide gels (sPAGE) from the RNA samples extracted from the chrysanthemum plants collected in regions of Artur Nogueira and Paranapanema, State of Sao Paulo, Brazil.
