# Supplementary material for: Surveys in the Chrysanthemum Production Areas of Brazil and Colombia Reveal That Weeds Are Potential Reservoirs of Chrysanthemum Stunt Viroid
Source: Viruses. 2019 Apr 17;11(4):355. doi: 10.3390/v11040355 (PMC6521175; doi:10.3390/v11040355)
Supplement: Supplementary file 1 [file viruses-11-00355-s001.pdf]

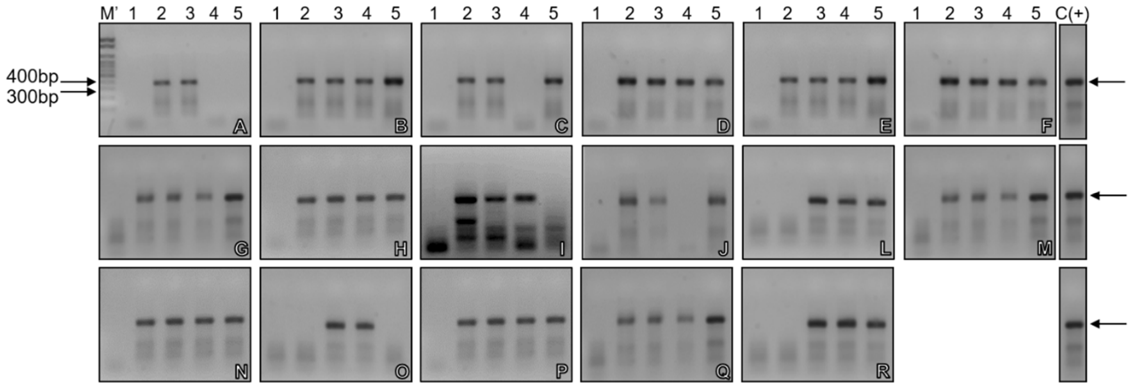

Figure S1. RT-PCR analysis to confirm the infectivity of the Brazilian chrysanthemum stunt viroid (CSVd) isolate in several inoculated hosts. Amplified DNA products were subjected to electrophoresis in 1.5% agarose gels, stained with ethidium bromide. The expected size of the PCR products went from 354 to 356 bp. M′, 100 bp DNA ladder; A, *Amaranthus viridis*; B, *Cardamine bonariensis*; C, *Chamaesyce hirta*; D, *Conyza bonariensis*; E, *Chrysanthemum carinatum*; F, *Digitaria sanguinalis*; G, *Emilia sagittata;* H, *Gomphrena globosa*; I, *Helianthus annuus*; J, *Lupinus polyphyllus*; L, *Mirabilis jalapa*; M, *Oxalis latifolia*; N, *Portulaca oleracea*; O, *Senecio cruentus*; P, *Solanum lycopersicum* "Rutgers"; Q, *Solanum tuberosum "*Agata"; R, *Catharanthus roseus*; 1, Mock-inoculated plants, 2-5, Inoculated plants; C(+), Positive control. Arrows indicate the expected size of the PCR products.

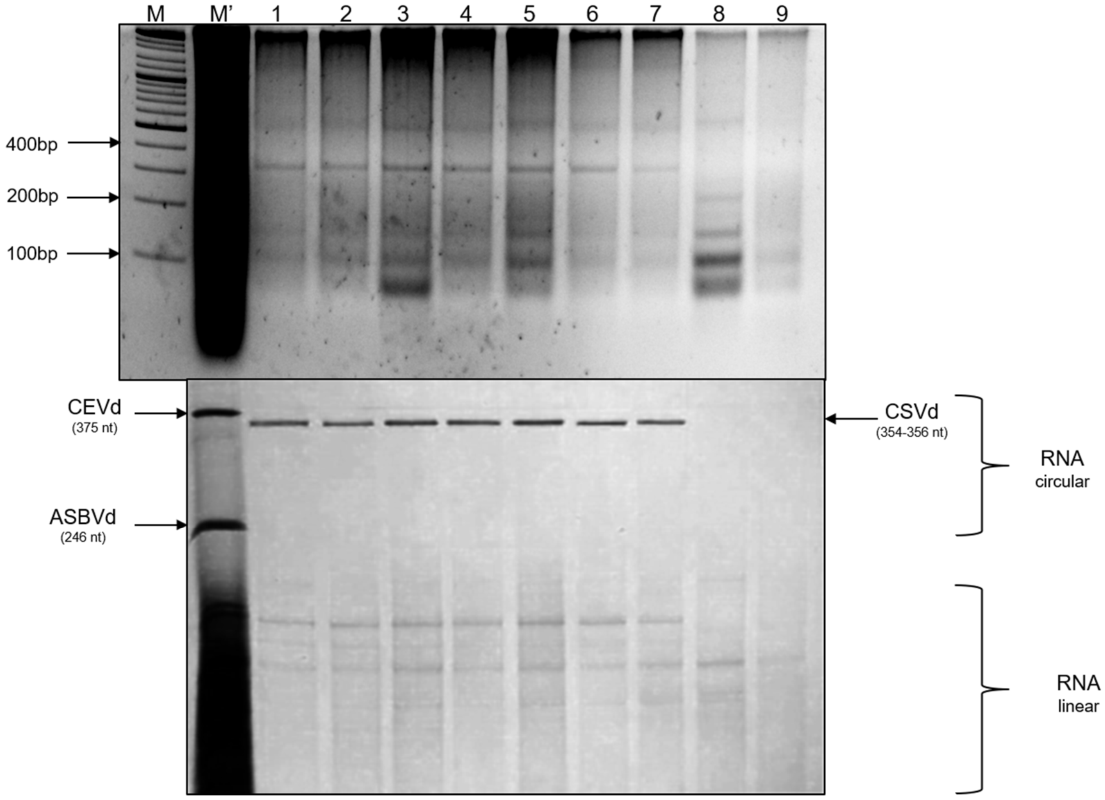

Figure S2. Sequential electrophoresis results on native and denaturing polyacrylamide gels (sPAGE) from the RNA samples extracted from the chrysanthemum plants collected in regions of Artur Nogueira, State of Sao Paulo, Brazil. M, 100 bp DNA ladder; M′, mixture of purified citrus exocortis viroid (CEVd) and avocado sunblotch viroid (ABSVd). RNAs extracted from infected tomato and avocado, respectively; 1 to 7, the RNA extracted from the infected chrysanthemum samples (2215-BR to 2221-BR). 8-9, samples of healthy chrysanthemum plants. The presence of circular RNAs with 354-

356 nucleotides (nt), corresponding to chrysanthemum stunt viroid (CSVd), is observed in the second gel.

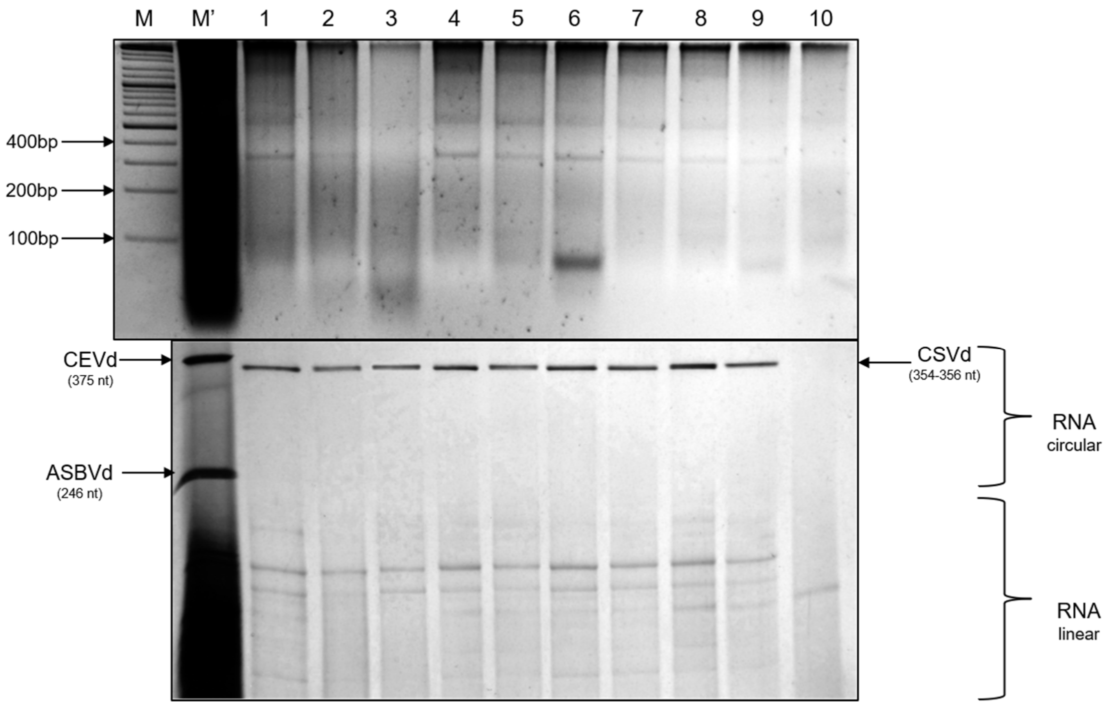

Figure S3. Sequential electrophoresis results on native and denaturing polyacrylamide gels (sPAGE) from the RNA samples extracted from the chrysanthemum plants collected in regions of Artur Nogueira and Paranapanema, State of Sao Paulo, Brazil. M, 100 bp DNA ladder; M ′, mixture of purified citrus exocortis viroid (CEVd) and avocado sunblotch viroid (ABSVd), The RNAs extracted from infected tomato and avocado, respectively; 1 to 9, the RNA extracted from the infected chrysanthemum samples (2222-BR to 2230-BR); 10, sample of a healthy chrysanthemum plant. The presence of circular RNA with 354-356 nucleotides (nt), corresponding to chrysanthemum stunt viroid (CSVd), is observed in the second gel.
